# 

**Published:** 1854-03

**Authors:** 


We have received from Messrs. Blanchard & Lea, copies of Bennett on the Uterus, Meigs on the Uterus, La Roche on Pneumonia and Malaria, and Fowne's Chemistry for Students, all of which will have mention in our next.

